# Increased Risk of Non-Hodgkin Lymphoma in Autoimmune Hepatitis: A Large Retrospective Cohort Study

**DOI:** 10.3390/jcm13206258

**Published:** 2024-10-20

**Authors:** Mifleh Tatour, Ziv Neeman, Ariel Aviv, Rawi Hazzan

**Affiliations:** 1 Clalit Health Services, Nof Hagalil 1710601, Israel; rawihazzan1@gmail.com; 2 Department of Family Medicine, Clalit Health Services, Afula 1710601, Israel; 3 Imaging Institute & Nuclear Medicine, Emek Medical Center, Clalit Health Services, Afula 183411, Israel; zivneeman@gmail.com; 4 Ruth and Bruce Rappaport, Faculty of Medicine, Technion—Institute of Technology, Haifa 3109601, Israel; ariel_av@clalit.org.il; 5 Hematology Unit, HaEmek Medical Center, Afula 183411, Israel; 6 Azrieli Faculty of Medicine, Bar-Ilan University, Safed 1311502, Israel; 7 Emek Medical Center, 21 Yitzhak Rabin Blvd, Afula 183411, Israel

**Keywords:** autoimmune hepatitis, non-hodgkin lymphoma, azathioprine, 6-mercaptopurine, mycophenolate mofetil

## Abstract

**Background/Objectives:** Autoimmune hepatitis (AIH) is a chronic inflammatory liver disease caused by an autoimmune attack on hepatocytes. The first-line treatment for AIH comprises two core components: glucocorticoids and thiopurine analog inhibitors and mycophenolate mofetil (MMF). Numerous studies have suggested an increased risk for lymphoma among patients with either rheumatoid arthritis or inflammatory bowel disease (IBD) who are treated with azathioprine/6-mercaptopurine (6-MP). The relative risk of non-Hodgkin lymphoma (NHL) among autoimmune hepatitis patients treated with these immunosuppressive drugs remains unclear. We aimed at investigating the risk of NHL across a large retrospective AIH cohort after a long-term follow-up. **Methods**: This retrospective, population-based study comprised approximately 2.7 million adults over two decades. It included adult patients aged 20 years or older at the time of autoimmune hepatitis diagnosis who had initiated treatment with azathioprine, 6-MP, or MMF. The primary outcome was the development of non-Hodgkin lymphoma. **Results:** The study initially included 834 patients diagnosed with AIH. A total of 685 patients remained in the research cohort after matching the data to the local cancer registry. Compared to the predicted NHL rate in the general population, NHL incidence was considerably higher in AIH patients (Standardized Incidence Ratio, SIR = 36.5). Subgroup studies showed that lymphoma mainly affected patients 45 years of age and over and was detected primarily during the first seven years following the AIH diagnosis. No correlation was found between the incidence of NHL and the treatment drug used. **Conclusions:** Patients with AIH exhibit a markedly higher risk of NHL compared to the general population.

## 1. Introduction 

Autoimmune hepatitis (AIH) is a chronic inflammatory liver disease caused by an autoimmune attack on hepatocytes characterized by circulating autoantibodies, hypergammaglobulinemia, and specific liver histological abnormalities [1]. Diagnosis is typically based on clinical, biochemical, serologic, and histological findings using the diagnostic scoring system provided by the International Autoimmune Hepatitis Group (IAIHG) [2,3].

Clinical manifestations of AIH range from asymptomatic disease to acute hepatitis and occasionally fulminant hepatic failure [4,5]. The first-line treatment for AIH comprises two core components: glucocorticoids (prednisone or budesonide) and azathioprine, leading to 80–90% remission [6]. The medication 6-mercaptopurine (6-MP) can serve as an alternative for patients who are intolerant to azathioprine [7]. Mycophenolate Mofetil (MMF) is employed in AIH patients who cannot tolerate azathioprine and 6-MP or those who show an unsatisfactory response to standard therapy [8]. Diagnosis of autoimmune hepatitis typically necessitates life-long treatment for most patients to prevent progression to cirrhosis and end-stage liver disease [9]. However, immunosuppression, either disease-mediated as in AIDS or iatrogenic as in the post-transplant state, is associated with an increased risk of lymphoma [10,11,12].

Lymphoma is a group of malignant neoplasms of lymphocytes with more than 90 subtypes. It is traditionally classified broadly as non-Hodgkin or Hodgkin lymphoma [13]. Non-Hodgkin lymphoma (NHL) is a blood-related malignancy originating from lymphocytes, accounting for 90% of all malignant lymphomas. [14] In 2020, NHL represented approximately 2.8% of the total cancer burden and contributed to 2.6% of all cancer-related mortality [15]. NHL risk factors include a range of demographics such as age, gender, race, ethnicity, geography, and family history [16,17]. Moreover, chemical and radiation exposure, whether environmentally or occupationally related, is also considered a risk factor for NHL [18,19]. The hazard ratio and incidence rates of NHL were also significantly higher in patients with hepatitis B or C compared to the general population, as reported by a recent nationwide cohort study from Taiwan [20]. Autoimmune diseases were also associated with an increased risk of NHL, as demonstrated in a large Swedish cohort study after a long-term follow-up. In this study, standardized incidence ratios (SIRs) significantly increased after multiple autoimmune diseases, mainly when these autoimmune diseases were diagnosed at younger ages. This association may be attributed to similarities in histological features between these conditions and NHL subtypes, or to shared origins between autoantibody-producing autoimmune diseases and histological NHL subtypes [21].

Regarding the risk of lymphoma in diseases treated with immunosuppressive therapy, a modestly increased risk was observed in patients with rheumatoid arthritis who were treated with azathioprine or 6-MP [22,23,24]. Furthermore, meta-analyses have consistently highlighted the increased risk of lymphoma in IBD patients treated with thiopurines such as azathioprine/6-MP. One meta-analysis reported an approximately fourfold increase in lymphoma risk among these patients [25]. At the same time, a more recent and comprehensive analysis indicated an even higher risk, nearly sixfold, compared to the general population [26]. Moreover, no study has provided an estimate of the relative risk of lymphoma among autoimmune hepatitis patients treated with these immunosuppressive drugs. To address the limitations in current knowledge and practice, we aimed at determining whether there is an increased risk of NHL in autoimmune hepatitis patients treated with azathioprine, 6-MP, or MMF within a large retrospective cohort study after a long-term follow-up.

## 2. Materials and Methods

Clalit Health Services (CHS) is Israel’s largest Health Maintenance Organization (HMO), catering to the healthcare needs of approximately 4.8 million members, constituting 52% of the Israeli population. It operates a network of 14 hospitals and around 1500 clinics spread nationwide, alongside laboratories, imaging facilities, and pharmacies. Integrating outpatient and inpatient data significantly enhances the precision and reliability of making diagnoses and selecting appropriate procedures. This retrospective, population-based study encompassed approximately 2.7 million adults and relied on data extracted from the comprehensive computerized CHD database. An electronic medical record (EMR) repository aggregates information from diverse sources, including records from primary care physicians, community specialty clinics, hospital admissions, laboratory tests, and pharmacy transactions. A registry of chronic disease diagnoses was compiled meticulously by employing diagnosis-specific algorithms, utilizing the International Classification of Diseases Ninth Revision (ICD-9) code framework. The earliest recorded date of diagnosis for autoimmune hepatitis (AIH) or lymphoma from any source was established as the defining date of diagnosis.

Patients with concurrent liver disease—viral hepatitis, primary biliary cholangitis, and primary sclerosing cholangitis—were excluded using chart diagnosis and ICD-9 code. Therefore, no patients with HBV or HCV were included as controls in this study. Furthermore, patients with concurrent autoimmune diseases or inflammatory bowel diseases (IBD) currently undergoing immunosuppressive therapy and individuals with a documented history of previous lymphoma were also excluded from this study. This exclusion was done to focus specifically on the effects of azathioprine, 6-mercaptopurine (6-MP), or mycophenolate mofetil (MMF) in treating autoimmune hepatitis while minimizing potential confounders related to prior lymphoma cases.

The standard treatment for AIH typically begins with administering Imuran (azathioprine) or second-line therapies combined with glucocorticoids or a two-week course of steroid monotherapy before initiating glucocorticoids-sparing drugs. Once biochemical remission is achieved, the steroid dose is gradually tapered until discontinuation, leaving the patient on glucocorticoid-sparing drugs. In our study, all patients initially received prednisone, but it was discontinued in all cases as part of their long-term treatment plan. Therefore, it was not feasible to isolate steroid use as a separate group for evaluating any potential increased risk of NHL. Consequently, our analysis focused on the prolonged use of azathioprine, 6-mercaptopurine, and MMF, excluding steroids from the evaluation of NHL risk.

To further ensure the accuracy and relevancy of our study, epidemiological data were obtained from the Israeli Cancer Registry, focusing on individuals aged 20 years and older between the years 2000 and 2020. This dataset includes a comprehensive breakdown of NHL cases by age categories (20–45, 45–64, and over 65) and gender subgroups to compare the incidence and distribution of non-Hodgkin lymphoma cases within our cohort to that of the general population. Consequently, our cohort selection was based on adult patients aged 20 years or older who had initiated treatment with azathioprine, 6-mercaptopurine (6-MP), or mycophenolate mofetil following the diagnosis of autoimmune hepatitis from 2000 to late 2020. Of note, none of the patients in the study had been diagnosed with AIH before 2000. These patients had clinical follow-ups extending to 2022.

Available data on sex, age, BMI at AIH diagnosis, liver histology, serological findings (antinuclear antibodies, smooth muscle antibodies, anti-liver kidney microsome type 1 antibodies), and serum IgG levels were extracted. Additional details collected included AIH treatment specifics (type of immunosuppression, dosage, starting date, and duration), laboratory results at AIH diagnosis every six months after that, dates of NHL diagnosis, and survival status. The baseline was set as the time point of AIH diagnosis, with the primary outcome being NHL development. Patient follow-up adhered to the standard practice of each center. Importantly, given the study’s retrospective nature, not all parameters were consistently available for every patient.

The study was conducted with the approval of the Helsinki Committee—Clalit, Health Services, Emek Medical Center, approval number 0091-23.

## 3. Statistical Analyses

Categorical variables were counted as percentages, while continuous variables were represented as means and standard deviations. Among the entire AIH cohort, the association between NHL incidence and intake of the three medications (azathioprine, 6-MP, and MMF) was performed separately using the Fisher’s exact test. The general population NHL rates were extracted from the Israeli cancer registry for 2010–2020 among males and females aged 20 or older. The NHL rates of the AIH patients and of the general population were compared using a standardized incidence ratio (SIR) with a 95% confidence interval (95%CI). 

Finally, a Kaplan–Meier curve was estimated with a 95% confidence interval for the main cohort of AIH patients, presenting the time to NHL for each follow-up year since the onset of AIH diagnosis.

Statistical analyses were performed using SAS 9.1 software (SAS Institute Inc., Cary, NC, USA). A *p*-value < 0.05 was considered statistically significant.

## 4. Results

**Figure 1:**
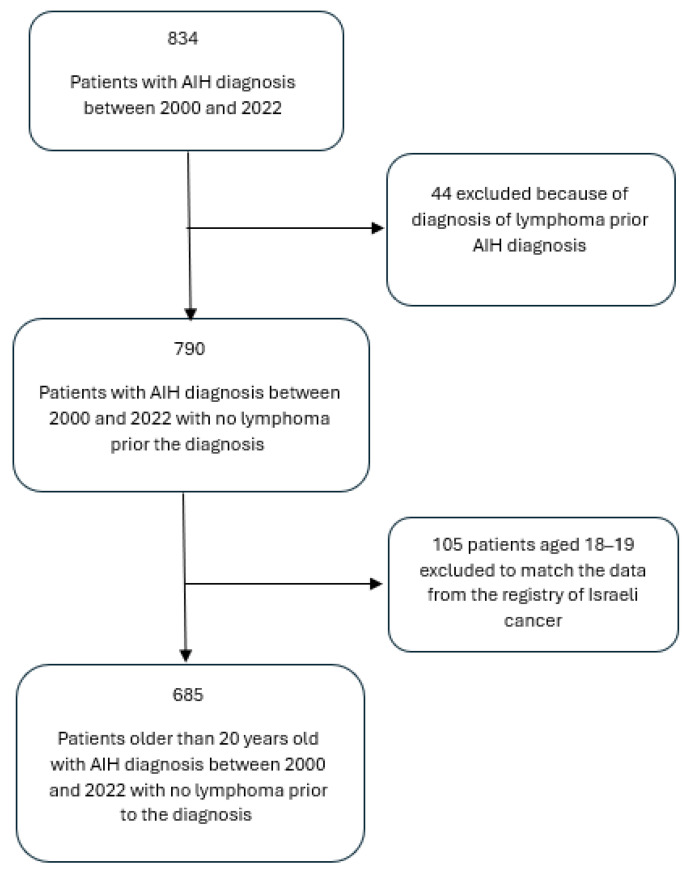
Patient flow chart. AIH, autoimmune hepatitis.

At the time of the data extraction, data on 834 patients with an initial diagnosis of autoimmune hepatitis (AIH) between 2000 and 2020 without concomitant liver or other autoimmune diseases or IBD who had received immunosuppressive therapy were retrieved from Clalit Medical Centers. Forty-four patients were excluded due to a prior lymphoma diagnosis, as well as 105 patients aged 18-19 in order to match the age categories used in the Israeli Cancer Registry, which are 0–19, 20–44, 45–64, and 65+. Since our study focused on patients aged 18 and older, we aligned both databases using the age groups 20–44, 45–64, and 65+. Consequently, the final sample size for our study was 685, as is shown in Figure 1.

Table 1 lists the baseline attributes of our final cohort, which included 685 patients diagnosed with AIH between 2000 and 2020. The mean age at diagnosis was 53.1 years, and most patients (over 83%) were female. A significant majority, 638 (93.1%) patients, were treated with azathioprine as a first-line treatment, and 127 (18.5%) patients used mycophenolate mofetil (MMF) as either first- or second-line therapy. In comparison, 41 (4.5%) patients also used 6-mercaptopurine (6-MP) as either first- or second-line therapy. The mean follow-up duration was 7.5 years. Most patients were treated for less than five years, but almost 30% received treatment for 5–10 years, and nearly 19% were treated for more than 10 years. Additionally, approximately 22% of the patients were diagnosed with cirrhosis at the time of AIH diagnosis. During the follow-up period, 17.1% of the patients died (Table 1).

Nine patients were diagnosed with NHL following AIH diagnosis, with all cases occurring in individuals aged 45 and older. Six of the nine cases of NHL happened in the 45–64 age group, representing two-thirds of the cases found in our study. Seven of the nine cases were females, representing over 80% of the AIH patient population diagnosed with NHL. Taking all the data together, the total standardized incidence ratio (SIR) for NHL in this cohort is 36.5 (Table 2).

Within the study cohort, 638 patients used azathioprine as their primary therapy, representing most of the cohort. OF these, 538 patients were treated exclusively with azathioprine, and six lymphoma cases were observed in this group, accounting for two-thirds of the lymphoma cases in our study. In this group, the mean duration from AIH diagnosis to lymphoma diagnosis was 5.59 years (range: 0.83 to 11.54 years). Seventy-five patients transitioned from azathioprine to mycophenolate mofetil (MMF), 14 transitioned to 6-mercaptopurine (6-MP), and 11 switched to MMF and 6-MP. One lymphoma case occurred in a patient who transitioned from azathioprine to MMF after approximately 1.5 years; no lymphoma cases were observed in the remaining transition categories. Additionally, 41 patients used MMF as their sole treatment, with two lymphoma cases identified in this group. No lymphoma cases were observed among the six patients who received 6-MP as their sole treatment (Table 3). Fisher’s exact test (*p*-value > 0.05) found no significant association between the use of specific medications and the incidence of lymphoma.

**Figure 2:**
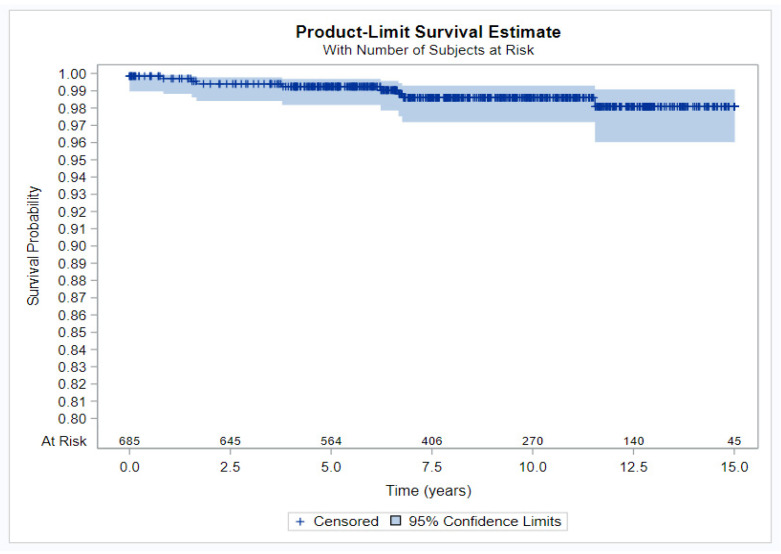
Kaplan–Meir curve.

Patients were followed for up to 15 years to monitor whether they remained lymphoma-free during treatment. As shown in Figure 2, a high survival rate for lymphoma was maintained throughout the follow-up years. Almost all lymphoma cases occurred in the first 6–7 years from diagnosis of AIH. Table 4 depicts the definitive rate of patients without lymphoma at each year of follow-up. A high survival rate was observed consistently throughout the follow-up period.

## 5. Discussion

In this pioneering study of nearly 700 autoimmune patients who were traced for a median of 7.5 years of follow-up, we found an SIR of 36.5—indicating a significantly increased risk of NHL in autoimmune patients treated with azathioprine, MMF, or 6-MP relative to the NHL rate expected in the general population. These findings are inconsistent with prior research demonstrating a modestly increased risk of lymphoma among IBD patients receiving these medications [25,26]. This heightened risk may alert clinicians and patients to the safety profile and potential complications associated with the disease’s clinical course and treatment.

Numerous Scandinavian studies have addressed the issue of increased risk of cancer in autoimmune hepatitis. A recent Danish nationwide cohort study observed an increased risk of incident cancers in autoimmune hepatitis [27]. Furthermore, a nationwide Swedish population-based cohort study documented a hazard ratio of 1.89 in developing lymphoma in patients with AIH [28]. Another high risk of developing lymphoma, with an SIR of 13.09, was also found in an additional large Swedish cohort study in AIH patients after a long-term follow-up [29]. These data may explain the significant risk of developing NHL in patients with AIH who were treated with immunosuppressive therapy. Thus, we hypothesize that the primary contribution to this increased lymphoma risk may be AIH’s intrinsic autoimmune nature. Various publications may legitimize this theory, two of which have shown that individuals with weakened immune systems, autoimmune disease, and certain infections are also at a higher risk of developing NHL [16,17]; others have shown that cancer incidence is higher in autoimmune disease (AD) patients [30,31,32,33,34,35,36,37,38]. Reinforcing this idea, multiple studies have demonstrated that the pro-tumorigenic effect observed in autoimmune diseases may stem from the chronic inflammation and immune dysregulation inherent to these conditions [39,40]. To address the potential influence of dosage on the observed differences in lymphoma development between inflammatory bowel disease (IBD) and autoimmune hepatitis (AIH), a comparison indicates that patients with inflammatory bowel disease (IBD) are typically treated with a recommended dose of azathioprine of 2.0–2.5 mg/kg/day [41]. In contrast, in AIH, the standard dosage of azathioprine is lower and varies slightly by region (50 to 150 mg daily in the United States and 1 to 2 mg/kg daily in Europe [42]), reinforcing the idea that the autoimmune pathology of AIH may be the main factor responsible for the heightened risk of NHL in AIH patients.

The medication 6-mercaptopurine (6-MP) can be used as an alternative for patients who are intolerant to azathioprine [43]. Data have shown that a combination of prednisone and MMF as a first-line treatment achieves higher remission of aminotransferase and IgG levels and a lower nonresponse rate in patients with AIH [44]. In our study, 127 patients received MMF either as a sole treatment or after transitioning from azathioprine, with three cases of NHL observed in this group, two of which were in patients receiving MMF as their sole treatment. Meanwhile, the primary treatment of azathioprine was associated with six lymphoma cases. This difference raises the question of whether MMF has a higher potential role in developing NHL compared to azathioprine. However, no statistically significant difference or elevated risk of NHL was found between patients treated with MMF and those treated with azathioprine. However, it is important to note that the available data are inconclusive due to the lack of extensive population studies and the need for extended follow-up periods. Therefore, further research is required, particularly given the increasing recognition of MMF as a potential first-line treatment for AIH.

Notably, no consensus exists on the optimal treatment duration of immunosuppressive drugs for patients with AIH [44,45]. Some data suggest that 80% of AIH patients achieve remission within three years with the current standard treatment regimen [46]. A minimum treatment duration of two years before considering treatment withdrawal has been proposed [44], while others suggest that a longer duration of immunosuppressive therapy is associated with lower relapse rates [47]. In our study, a minority of patients continued therapy for extended periods, possibly due to achieving remission, while others may have died during follow-up. Notably, nearly all patients who developed NHL did so within the first seven years of AIH diagnosis, with a mean time from AIH diagnosis to NHL development of approximately six years in those primarily treated with azathioprine. This finding suggests that the first years of AIH treatment may be crucial for monitoring the development of NHL. However, these observations require validation through more extensive multicenter studies with possible lymphoma incidence and more extended follow-up periods to provide a clearer understanding and confirmation.

Our research findings provide comprehensive insights into the relationship between NHL and autoimmune hepatitis, featuring strengths and limitations. The study benefits from a large dataset comprising a substantial number of patients with autoimmune hepatitis receiving these medications, allowing for more profound statistical analysis and potentially enhancing the reliability of the results. Additionally, this study’s longitudinal design enables tracing patients over an extended period of time; patients were traced for a median of nearly eight years following diagnosis, offering a glimpse into the long-term outcomes and potential adverse effects associated with treatment. However, the retrospective nature of the research poses a known limitation, as it may include biases and confounding factors that could influence the interpretation of the results. Moreover, our study primarily involves a single-country population, raising concerns about the generalizability of findings to patients of different ethnic backgrounds. Additional prospective multicenter studies conducted globally are warranted to validate and extend these findings, ensuring their applicability across various patient populations and healthcare settings.

## Figures and Tables

**Table 1:** Baseline attributes of the Final cohorts.

Total number of AIH patients	685
Mean age at the diagnosis (years ± SD)	53.1 ± 16.7
Female (%)	83.5%
Male (%)	16.5%
BMI at diagnosis (BMI ± SD)	27.1 + 5.5
Treated with azathioprine (%)	638 (93.1%)
Treated with MMF (%)	127 (18.5%)
Treated with Purinethol/ 6-MP (%)	31 (4.5%)
Mean follow-up (years ± SD)	7.5 ± 4.5
Years of treatment (% patients)	
0–5 years	51.7%
5–10 years	29.6%
More than 10 years	18.7%
Cirrhosis at diagnosis (%patients)	21.9%
Death during follow-up (death, %death)	117 (17.1%)

AIH, autoimmune hepatitis; MMF, mycophenolate mofetil; 6-MP, 6-mercaptopurine; BMI, body mass index; SD, standard deviation.

**Table 2:** Subdivision of the data based on age and gender.

Gender	Age	Sample Size AIH (2000–2020)	Rate (per 100,000)	Expected Cases	Observed Cases
Male	20–44	41	6.42	0.003	0
	45–64	40	32.71	0.013	1
	65+	32	98.56	0.032	1
Female	20–44	179	5.32	0.010	0
	45–64	239	28.01	0.067	5
	65+	154	79.73	0.123	2
	Total	685		0.247	9

SIR = 36.5 [95%CI: 16.55–64.25]. The confidence interval of the SIR values outside 1.0 is considered statistically significant. SIR, standardized incidence ratio (expected cases/observed cases).

**Table 3:** Statistical comparison between the therapies in developing lymphoma.

Combination of Therapies Used	Number of Patients (N = 685)	Number of Lymphoma Cases (N = 9)	No of Patients without Lymphoma (N = 676)	Statistical Significance (*p* Value)	Meantime in Years from AIH Diagnosis to Lymphoma Diagnosis (min, max)
Azathioprine	538	6 (66.7%)	532 (78.7%)	0.4125	5.59 (0.8–11.54)
MMF	41	2 (22.2%)	39 (5.8%)	0.0963	1.43 (1.2–1.66)
6-MP	6	0 (0%)	6 (0.9%)	>0.99	N/A
Azathioprine à MMF	75	1 (11.1%)	74 (10.9%)	>0.99	3.78
Azathioprine à 6-MP	14	0 (0%)	14 (2.1%)	>0.99	N/A
Azathioprine à MMF à 6-MP	11	0 (0%)	11 (1.6%)	>0.99	N/A

N/A: not applicable.

**Table 4:** Rate of lymphoma-free patients during follow-up.

FU	Survival	95%CI	
		Low	Up
0–1	99.70%	98.82%	99.93%
1–2	99.40%	98.41%	99.77%
2–3	99.40%	98.41%	99.77%
3–4	99.24%	98.18%	99.68%
4–5	99.24%	98.18%	99.68%
5–6	99.24%	98.18%	99.68%
6–7	98.60%	97.18%	99.30%
7–8	98.60%	97.18%	99.30%
8–9	98.60%	97.18%	99.30%
9–10	98.60%	97.18%	99.30%
10–11	98.60%	97.18%	99.30%
11–12	98.08%	96.03%	99.08%

FU, follow-up; CI, confidence interval.

## Data Availability

The datasets generated and/or analyzed during the current study are not Publicly available because of clalit health service policy but are available from the corresponding author on reasonable request.

## Abbreviations

AIH, autoimmune hepatitis; IBD, inflammatory bowel disease; NHL, non-Hodgkin lymphoma; MMF, mycophenolate mofetil; 6-MP, 6-mercaptopurine; AD, autoimmune disease; BMI, body mass index; SD, standard deviation; SIR, standardized incidence ratio.

## References

[1] Mieli-Vergani G., Vergani D., Czaja A.J., Manns M.P., Krawitt E.L., Vierling J.M., Lohse A.W., Montano-Loza A.J. (2018). Autoimmune hepatitis. Nat. Rev. Dis. Primers.

[2] Hennes E.M., Zeniya M., Czaja A.J., Parés A., Dalekos G.N., Krawitt E.L., Bittencourt P.L., Porta G., Boberg K.M., Hofer H. (2008). Simplified criteria for the diagnosis of autoimmune hepatitis. Hepatology.

[3] European Association for the Study of the Liver (2015). EASL Clinical Practice Guidelines: Autoimmune hepatitis. J. Hepatol..

[4] Zachou K., Muratori P., Koukoulis G.K., Granito A., Gatselis N., Fabbri A., Dalekos G.N., Muratori L. (2013). Review article: Autoimmune Hepatitis—Current management and challenges. Aliment. Pharmacol. Ther..

[5] Vierling J.M. (2015). Autoimmune Hepatitis and Overlap Syndromes: Diagnosis and Management. Clin. Gastroenterol. Hepatol..

[6] Ramamoorthy S., Cidlowski J.A. (2016). Corticosteroids: Mechanisms of action in health and disease. Rheum. Dis. Clin. N. Am..

[7] Hübener S., Oo Y.H., Than N.N., Hübener P., Weiler-Normann C., Lohse A.W., Schramm C. (2016). Efficacy of 6-mercaptopurine as second-line treatment for patients with autoimmune hepatitis and azathioprine intolerance. Clin. Gastroenterol. Hepatol..

[8] Mack C.L., Adams D., Assis D.N., Kerkar N., Manns M.P., Mayo M.J., Vierling J.M., Alsawas M., Murad M.H., Czaja A.J. (2020). Diagnosis and management of autoimmune hepatitis in adults and children: 2019 practice guidance and guidelines from the American Association for the Study of Liver Diseases. Hepatology.

[9] Pape S., Schramm C., Gevers T.J. (2019). Clinical management of autoimmune hepatitis. United Eur. Gastroenterol. J..

[10] Kinlen L.J., Schottenfeld Fraumeni D. (1996). Immunologic factors, including AIDS. Cancer Epidemiology and Prevention.

[11] Opelz G., Henderson R. (1993). Incidence of non-Hodgkin lymphoma in kidney and heart transplant recipients. Lancet.

[12] Wilkinson A.H., Smith J.L., Hunsicker L.G., Tobacman J., Kapelanski D.P., Johnson M., Wright F.H., Behrendt D.M., Corry R.J. (1989). Increased frequency of posttransplant lymphomas in patients treated with cyclosporine, azathioprine, and prednisone. Transplantation.

[13] Lewis W.D., Lilly S., Jones K.L. (2020). Lymphoma: Diagnosis and Treatment. Am. Fam. Physician.

[14] Sapkota S., Shaikh H. (2021). Non-Hodgkin Lymphoma.

[15] Observatory G.C. Fact Sheet: Non-Hodgkin Lymphoma. https://gco.iarc.fr/today/data/factsheets/cancers/34-Non-hodgkin-lymphoma-factsheet.pdf.

[16] Chiu B.C., Weisenburger D.D., Zahm S.H., Cantor K.P., Gapstur S.M., Holmes F., Burmeister L.F., Blair A. (2004). Agricultural pesticide use, familial cancer, and risk of non-Hodgkin lymphoma. Cancer Epidemiol. Biomark. Prev..

[17] Chang E.T., Smedby K.E., Zhang S.M., Hjalgrim H., Melbye M., Ost A., Glimelius B., Wolk A., Adami H.-O. (2005). Dietary factors and risk of non-Hodgkin lymphoma in men and women. Cancer Epidemiol. Biomark. Prev..

[18] Skibola C.F. (2007). Obesity, diet and risk of non-Hodgkin lymphoma. Cancer Epidemiol. Biomark. Prev..

[19] Kane E., Skibola C.F., Bracci P.M., Cerhan J.R., Costas L., Smedby K.E., Holly E.A., Maynadié M., Novak A.J., Lightfoot T.J. (2015). Non-Hodgkin lymphoma, body mass index, and cytokine polymorphisms: A pooled analysis from the InterLymph consortium. Cancer Epidemiol. Biomark. Prev..

[20] Lai Y.-R., Chang Y.-L., Lee C.-H., Tsai T.-H., Huang K.-H., Lee C.-Y. (2022). Risk of Non-Hodgkin Lymphoma among Patients with Hepatitis B Virus and Hepatitis C Virus in Taiwan: A Nationwide Cohort Study. Cancers.

[21] Fallah M., Liu X., Ji J., Försti A., Sundquist K., Hemminki K. (2014). Autoimmune diseases associated with non-Hodgkin lymphoma: A nationwide cohort study. Ann Oncol..

[22] Silman A.J., Petrie J., Hazleman B., Evans S.J. (1988). Lymphoproliferative cancer and other malignancy in patients with rheumatoid arthritis treated with azathioprine: A 20 year follow up study. Ann. Rheum. Dis..

[23] Kinlen L.J. (1985). Incidence of cancer in rheumatoid arthritis and other disorders after immunosuppressive treatment. Am. J. Med..

[24] Asten P., Barrett J., Symmons D. (1999). Risk of developing certain malignancies is related to duration of immunosuppressive drug exposure in patients with rheumatic diseases. J. Rheumatol..

[25] Kandiel A., Fraser A.G., Korelitz B.I., Brensinger C., Lewis J.D. (2005). Increased risk of lymphoma among inflammatory bowel disease patients treated with azathioprine and 6-mercaptopurine. Gut.

[26] Kotlyar D.S., Lewis J.D., Beaugerie L., Tierney A., Brensinger C.M., Gisbert J.P., Loftus E.V., Peyrin-Biroulet L., Blonski W.C., Van Domselaar M. (2015). Risk of lymphoma in patients with inflammatory bowel disease treated with azathioprine and 6-mercaptopurine: A meta-analysis. Clin. Gastroenterol. Hepatology..

[27] Jensen M.D., Jepsen P., Vilstrup H., Grønbæk L. (2022). Increased Cancer Risk in Autoimmune Hepatitis: A Danish Nationwide Cohort Study. Am. J. Gastroenterol..

[28] Sharma R., Verna E.C., Simon T.G., Söderling J., Hagström H., Green P.H.R., Ludvigsson J.F. (2022). Cancer Risk in Patients With Autoimmune Hepatitis: A Nationwide Population-Based Cohort Study With Histopathology. Am. J. Epidemiol..

[29] Werner M., Almer S., Prytz H., Lindgren S., Wallerstedt S., Björnsson E., Bergquist A., Sandberg-Gertzén H., Hultcrantz R., Sangfelt P. (2009). Hepatic and extrahepatic malignancies in autoimmune hepatitis. A long-term follow-up in 473 Swedish patients. J. Hepatol..

[30] Hemminki K., Liu X., Ji J., Sundquist J., Sundquist K. (2012). Effect of autoimmune diseases on mortality and survival in subsequent digestive tract cancers. Ann. Oncol..

[31] Hemminki K., Liu X., Ji J., Sundquist J., Sundquist K. (2012). Kaposi sarcoma and Merkel cell carcinoma after autoimmune disease. Int. J. Cancer.

[32] Hemminki K., Liu X., Forsti A., Ji J., Sundquist J., Sundquist K. (2013). Subsequent leukaemia in autoimmune disease patients. Br. J. Haematol..

[33] Liu X., Ji J., Forsti A., Sundquist K., Sundquist J., Hemminki K. (2013). Autoimmune disease and subsequent urological cancer. J. Urol..

[34] Hemminki K., Liu X., Ji J., Försti A., Sundquist J., Sundquist K. (2012). Effect of autoimmune diseases on risk and survival in female cancers. Gynecol. Oncol..

[35] Hemminki K., Liu X., Ji J., Sundquist J., Sundquist K. (2012). Effect of autoimmune diseases on risk and survival in histology-specific lung cancer. Eur. Respir. J..

[36] Hemminki K., Liu X., Forsti A., Ji J., Sundquist J., Sundquist K. (2012). Effect of autoimmune diseases on incidence and survival in subsequent multiple myeloma. J. Hematol. Oncol..

[37] Goldin L.R., Landgren O. (2009). Autoimmunity and lymphomagenesis. Int. J. Cancer.

[38] Landgren A.M., Landgren O., Gridley G., Dores G.M., Linet M.S., Morton L.M. (2011). Autoimmune disease and subsequent risk of developing alimentary tract cancers among 4.5 million US male veterans. Cancer.

[39] Moss S.F., Blaser M.J. (2005). Mechanisms of disease: Inflammation and the origins of cancer. Nat. Clin. Pract. Oncol..

[40] Grivennikov S.I., Greten F.R., Karin M. (2010). Immunity, inflammation, and cancer. Cell.

[41] Fraser A.G., Orchard T.R., Jewell D.P. (2002). The efficacy of azathioprine for the treatment of inflammatory bowel disease: A 30 year review. Gut.

[42] Goel A., Kwo P. (2024). Treatment of Autoimmune Hepatitis. Clin. Liver Dis..

[43] Yu Z.J., Zhang L.L., Huang T.T., Zhu J.S., He Z.B. (2019). Comparison of mycophenolate mofetil with standard treatment for autoimmune hepatitis: A meta-analysis. Eur. J. Gastroenterol. Hepatol..

[44] Manns M.P., Czaja A.J., Gorha J.D., Krawitt E.L., Mieli-Vergani G., Vergani D., Vierling J.M., American Association for the Study of Liver Diseases (2010). Diagnosis and management of autoimmune hepatitis. Hepatology.

[45] Gleeson D., Heneghan M.A. (2011). British Society of Gastroenterology (BSG) guidelines for management of autoimmune hepatitis. Gut.

[46] Lamers M.M., van Oijen M.G., Pronk M., Drenth J.P. (2010). Treatment options for autoimmune hepatitis: A systematic review of randomized controlled trials. J. Hepatol..

[47] Kanzler S., Gerken G., Löhr H., Galle P.R., Meyer zum Büschenfelde K.H., Lohse A.W. (2001). Duration of immunosuppressive therapy in autoimmune hepatitis. J. Hepatol..

